# Systematic review of available evidence on 11 high-priced inpatient orphan drugs

**DOI:** 10.1186/1750-1172-8-124

**Published:** 2013-08-16

**Authors:** Tim A Kanters, Caroline de Sonneville-Koedoot, W Ken Redekop, Leona Hakkaart

**Affiliations:** 1 Institute for Medical Technology Assessment, Department of Health Policy & Management, Erasmus University Rotterdam, Burgemeester Oudlaan 50, P.O. Box 1738, 3000DR Rotterdam, The Netherlands

**Keywords:** Rare diseases, Orphan drugs, Evidence based medicine, Clinical effectiveness, Cost-effectiveness, Budget impact

## Abstract

**Background:**

Attention for Evidence Based Medicine (EBM) is growing, but evidence for orphan drugs is argued to be limited and inferior. This study systematically reviews the available evidence on clinical effectiveness, cost-effectiveness and budget impact for orphan drugs.

**Methods:**

A systematic review was performed in PubMed, Embase, NHS EED and HTA databases for 11 inpatient orphan drugs listed on the Dutch policy rule on orphan drugs. For included studies, we determined the type of study and various study characteristics.

**Results:**

A total of 338 studies met all inclusion criteria. Almost all studies (96%) focused on clinical effectiveness of the drug. Of these studies, most studies were case studies (41%) or observational studies (39%). However, for all orphan diseases at least one experimental or quasi-experimental study was found, and a randomized clinical trial was available for 60% of the orphan drugs. Eight studies described the cost-effectiveness of an orphan drug; an equal number described an orphan drug’s budget impact.

**Conclusions:**

Despite the often heard claim that RCTs are not feasible for orphan drugs, we found that an RCT was available in 60% of orphan drugs investigated. Cost-effectiveness and budget impact analyses for orphan drugs are seldom published.

## Background

Evidence based medicine (EBM) stresses the importance of evidence stemming from clinical studies next to clinical experience, rather than making treatment decisions on intuition and clinical experience alone [1]. Attention for EBM in reimbursement decisions on drugs has grown over the years, also for policy decisions concerning drugs for rare diseases (i.e. orphan drugs). Compared to the evidence base for common drugs, however, the available evidence on effectiveness, cost-effectiveness and budget impact of most orphan drugs is limited. The evidence that is available, often does not meet traditional quality standards. Small number of patients and heterogeneity of the diseases hampers enrollment of sufficient patients in trials [2,3]. Randomized controlled trials (RCTs) are therefore often claimed to be unsuitable for orphan diseases [4,5] and thus inferior evidence is deemed satisfactory by decision makers [6,7].

In the Netherlands, from 2006 till 2012 a policy rule enabled temporary reimbursement for orphan drugs. For these drugs, treatment centers are required to collect data on effectiveness and cost-effectiveness over a period of 4 years. The Dutch policy rule on orphan drugs lists 11 inpatient therapies for 10 orphan diseases (Table 1); two therapies are listed on the policy rule for one indication (Fabry disease). Table 1 also provides the number of patients deemed eligible for treatment in the Netherlands as well as the estimated costs per patient and total budget impact of the treatment and the year for which these figures were estimated. Finally, Table 1 provides information about whether the EMA provided marketing authorization under exceptional circumstances (i.e. authorization was granted despite an incomplete dossier with respect to safety and/or efficacy). These estimates were based on the Dutch temporary reimbursement dossiers.

**Table 1:** Orphan drugs and indications on the Dutch policy rule

**Indication**	**Treatment**	Prevalence (100,000 live births)*	Estimated annual costs / patient in € **	Dutch budget impact in € **	**Year costs were assessed**	**Exceptional circumstances authorization**
Acute Lymphoid Leukemia (ALL) in children	Clofarabine (Evoltra)	8.1	54 K	0.7 M	2007	Yes
Cryopyrin Associated Periodic Syndromes (CAPS)	Canakinumab (Ilaris)	0.10 ***	66 K	2.6 M	2010	Yes
Chronic Lymphoid Leukemia (CLL) in patients refractory to fludarabine and alemzumab	Ofatumumab (Arzerra)	30	39 K	1.0 M	2011	No
Fabry disease	Agalsidase α (Replagal)	30	199 K	3.2 M	2009	Yes
Fabry disease	Agalsidase β (Fabrazyme)	30	194 K	5.7 M	2009	No
Mucopolysaccharidosis I (MPSI)	Laronidase (Aldurazyme)	1.3	300 K	7.5 M	2003	Yes
Mucopolysaccharidosis II (MPSII)	Idursulfase (Elaprase)	0.6	600 K	9.6 M	2007	Yes
Mucopolysaccharidosis VI (MPSVI)	Galsulfase (Naglazyme)	0.16	600 K	4.2 M	2007	Yes
Paroxysmal Nocturnal Hemoglobinuria (PNH)	Eculizumab (Soliris)	0.8	358 K	10.6 M *****	2008-2010	No
Pompe disease	Alglucosidase alfa (Myozyme)	1.5	38 K; 382 K ****	31.0 M	2007	No
Soft Tissue Sarcoma (STS)	Trabectedin (Yondelis)	23.7	22 K	5.3 M	2010	Yes

* European estimates obtained from Orphanet [8]; ** Obtained from CFH (accessible through http://www.cvz.nl, in Dutch); *** U.S. estimate [9]; **** Costs for infantile patients are estimated at €38 K, and costs for adults are estimated at €382 K ***** Average estimated yearly budget impact for the period 2008-2010.

In this study, we examine what evidence on effectiveness, cost-effectiveness and budget impact is available for these drugs. For this purpose, a systematic review was conducted. The findings of this review provide insight into what type of studies (with respect to study design) were performed in orphan diseases despite small numbers of patients.

## Methods

### Search strategy

The PRISMA guidelines for systematic reviews were followed [10]. A literature search was conducted in PubMed and Embase for all orphan drugs on the policy rule. The literature searches were performed in August 2012. The search terms included the disease and treatment (brand name and generic name; Table 1). For instance the following search string was performed for acute lymphoid leukemia: Acute Lymphoid Leukemia [OR] Acute Lymphatic Leukemia [AND] Clofarabine [OR] Evoltra. Similar search strings were used for the other orphan drugs. In both PubMed and Embase, the search was limited to English results. No additional search terms or limitations were used to ensure all relevant studies were found. A supplementary search was performed in the NHS Economic Evaluation Database (NHS EED) and HTA database to identify literature on cost-effectiveness (both databases were accessed through http://www.crd.york.ac.uk/CRDWeb/HomePage.asp). Only treatment (brand name and generic name) was used as a search term. The searches in the grey literature were performed in September 2012.

### Study selection

Eligibility criteria were stated before assessment of the studies. Two researchers (TK & CdS) subsequently decided independently on selection of the studies. In the first selection round, studies from PubMed and Embase were screened on the basis of titles and abstracts. In a consensus meeting differences were discussed. The same reviewers then assessed the full-text articles of the remaining studies. Studies from grey literature were assessed in the same way.

The following eligibility criteria were used for all drugs investigated (in this order):

1. Relevance to disease of interest: only studies on the disease of interest were included (e.g. exclude studies on the treatment of other diseases, orphan diseases in general, etc.)

2. Primary focus on disease of interest: only studies focusing primarily on the disease of interest were included (e.g. exclude studies on multiple diseases one of which is the disease of interest)

3. Treatment: only studies on the effectiveness, cost-effectiveness and budget impact of the treatment of interest were included (e.g. exclude studies on alternative treatment options)

4. Treatment of humans: only studies on the treatment of humans were included

5. Format: only research articles were included (e.g. exclude conference proceedings, editorials, comments, letters, conference abstracts, supplements etc.)

6. Summaries: Only original research articles were included (e.g. exclude summaries and reviews)

7. Duplicates: Multiple publications on the same study were identified in the assessment of full-texts. These studies were included in the analyses only once, to avoid double counting.

8. Accessibility: papers for which no abstract and no full-text were available were excluded.

For Acute Lymphoid Leukemia (ALL) and Chronic Lymphoid Leukemia (CLL), additional exclusion criteria were defined. When reviewing the evidence for ALL, studies in adult patient populations were excluded, as the treatment is only registered for the use in children and adolescents in the Netherlands. For CLL, only studies on patients who were refractory to fludarabine and alemtuzumab were included; studies on ofatumumab in previously untreated patients were also excluded to comply with the Dutch indication for of atumumab. When the drug was provided as part of a combination therapy, the record was excluded on account of the third exclusion criterion (i.e. the focus was not on the treatment of interest).

### Analyses of included studies

Two researchers (TK and CdS) carried out the data extraction independently. Included studies were classified into three groups with respect to subject: 1) effectiveness; 2) budget impact; 3) cost-effectiveness. Studies could potentially be classified into more than one group.

Studies on clinical effectiveness were examined using the hierarchical level of the study design [11]. Interventional studies (experimental or quasi-experimental designs) were defined as studies in which the investigator determined treatment regimen. In contrast, in observational studies treatment was decided on the basis of clinical characteristics of individual patients. Studies were labeled case studies when patients were individually described. Extension studies of clinical trials were considered as observational studies when all patients were treated with the new drug.

In addition, several key elements of study design were identified: control, randomization, blinding, follow-up duration and number of patients enrolled in the study. Controls, if any, could be placebo, historical, healthy and untreated patients or patients receiving supportive care. Randomization could have been done with respect to treatment, dosage, route of administration or no randomization. Blinding options were double blind, investigator blind, patient blind or open label. For interventional studies the follow-up duration of the study was also assessed.

Articles were considered as describing budget impact when total costs on a societal or health care level were provided; the sole mention of treatment cost per patients was not considered as a budget impact analysis. The relationship between disease prevalence and available evidence was tested using a Spearman correlation.

## Results

**Figure 1:**
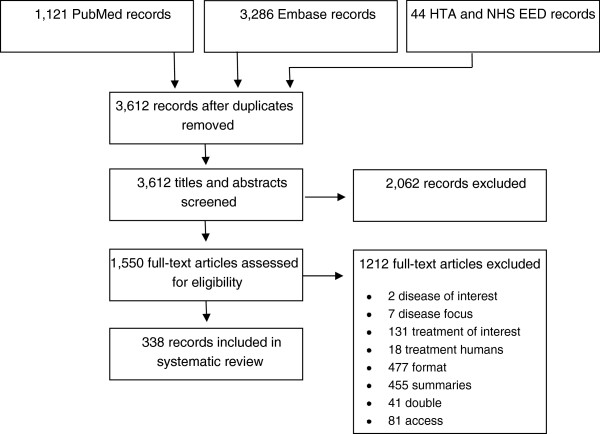
Flow chart of inclusion of studies.

Figure 1 shows that Embase yielded the most results from the literature. Searches in grey health economic literature resulted through the HTA and NHS EED databases in another 44 studies. After removal of duplicates, 3,612 unique studies remained. A total of 2,062 studies were excluded after screening titles and abstracts. Consequently, 1,550 (42.9% of total studies assessed) full-text articles were assessed, of which 338 met the inclusion criteria. More than one third of the 1,550 articles (39.4%) were excluded on the basis of format and a similar number (37.5%) was excluded due to being summary articles.

Table 2 shows that almost all of the 338 included studies described effectiveness of therapy. Only eight studies focused on cost-effectiveness, two of which were found in published papers and six in the grey literature (i.e. reports from various national health technology assessment agencies). Eight studies described the budget impact of an orphan drug. Budget impact for agalsidase α and agalsidase β was described in the same article. Six reports from the grey literature described both cost-effectiveness and budget impact of an orphan drug.

**Table 2:** Subject of included articles

	**Included studies**	**Effectiveness**	**Cost-effectiveness**	**Budget impact**
ALL	8	7	1	1
CAPS	7	7	0	0
CLL	3	2	1	0
Fabry (agalsidase α)	65	63	0	2
Fabry (agalsidase β)	66	64	1	1
MPSI	26	26	0	0
MPSII	22	21	1	1
MPSVI	24	24	0	0
PNH	23	21	1	2
Pompe	53	53	0	0
STS	41	38	3	1
Total	338	326	8	8

Six studies described both cost-effectiveness and budget impact; Six studies were included both for agalsidase α and agalsidase β; Six studies were included for multiple MPS diseases; In ten articles (one for CAPS; four for Fabry (α); one for Fabry (β); one for MPSI and three for Pompe) more than one study was described.

### Effectiveness

Case studies made up the largest share (42%) of all studies on effectiveness (see Tables 3 and 4). A further 39% included studies described observational studies. Although only a limited number of interventional studies were described (in total 19%), Table 3 shows that interventional studies were available for all drugs. For most interventional studies, study designs were adapted, particularly with respect to use of a placebo control group and blinding. Only a minor share of interventional studies (n = 14; 22%) involved a randomized, placebo-controlled, double blinded clinical trial. For four orphan drugs, no such study was available.

**Table 3:** Study characteristics of interventional studies

	**Number (% of all effectiveness studies)**	**Control group**	**Random-ization**	**Open label**	RCT*	**Single center**	**International**	**Mean follow-up duration in weeks [min-max]**	**Mean number of patients [min-max]**
ALL	2 (29%)	0	0	2	0	1	0	Unknown	39 [17-61]
CAPS	2 (22%)	1	1	1	1	0	2	64 [24-104]	99 [31-166]
CLL	2 (100%)	0	0	2	0	0	2	78 [52-104]	86 [33-138]
Fabry (α)	15 (22%)	8	8	7	6	4	5	35 [10-104]	26 [10-80]
Fabry (β)	10 (16%)	4	3	8	2	2	5	85 [20-234]	50 [13-134]
MPSI	3 (12%)	2	2	2	1	0	3	35 [26-52]	33 [20-45]
MPSII	3 (14%)	2	2	1	2	0	1	44 [26-53]	39 [10-96]
MPSVI	3 (13%)	0	1	2	0	0	2	95 [48-190]	8 [7-10]
PNH	4 (19%)	1	1	3	1	1	2	26 [12-52]	56 [11-97]
Pompe	8 (14%)	3	2	7	1	3	4	70 [26-120]	23 [5-90]
STS	12 (32%)	0	1	12	0	0	6	42 [9-104]	64 [13-270]
Total	64 (19%)	21	21	47	14	11	32	57 [9-234]	48 [7-270]

*RCT = Placebo controlled, randomized, double-blind, clinical trial.

**Table 4:** Study characteristics of observational studies and case studies

	**Observational studies**					**Case studies**
	**Number (% of all effectiveness studies)**	**Control group**	**Single center**	**Registry data**	**Mean number of patients [min-max]**	**Number (% of all effectiveness studies)**
ALL	1 (14%)	0	0	0	5 [5]	4 (57%)
CAPS	5 (56%)	3	2	0	25 [10-35]	2 (22%)
CLL	0 (0%)	-	-	-	-	0 (0%)
Fabry (α)	37 (55%)	10	17	13	102 [7-752]	15 (22%)
Fabry (β)	29 (45%)	13	18	3	62 [6-822]	25 (39%)
MPSI	7 (27%)	0	5	1	141 [5-891]	16 (62%)
MPSII	9 (41%)	1	3	3	56 [11-124]	10 (45%)
MPSVI	5 (21%)	0	1	1	41 [3-132]	16 (67%)
PNH	8 (38%)	5	0	0	58 [6-187]	9 (43%)
Pompe	14 (25%)	2	4	0	26 [8-74]	34 (61%)
STS	17 (45%)	3	8	0	60 [7-379]	9 (24%)
Total	132 (39%)	37	58	21	58 [3-891]	140 (42%)

In 33% of the interventional studies and 28% of the observational studies a control group was used. In most studies healthy controls and historical controls were used as a comparison group; in only 21% (12/58) of the studies the control group received placebo treatment.

Interventional studies often applied multicenter and international study designs to enlarge sample sizes. Nevertheless, the size of the study population in these interventional studies averaged 48 patients (range 7-270). A total of 16% of observational studies (21/132) were based on registry data; the majority stemming from the registry set up for agalsidase α in Fabry disease.

No significant correlation was found between the disease prevalence and the number of included studies on effectiveness (rho = 0.373; p = 0.258). There appeared to be fewer studies available for orphan drugs with market authorization under exceptional circumstances compared to drugs authorized without exceptional circumstances (average 25 versus 36 studies, respectively). However, the limited number of drugs under study limited the statistical power to detect any important differences.

### Cost-effectiveness analyses and budget impact studies

Only eight studies described a cost-effectiveness analysis of an orphan drug. All cost-effectiveness analyses made use of a health economic model. Seven studies presented a cost-utility analysis, in which incremental costs per QALY (quality adjusted life years) ratio was calculated, whereas one study described a cost per life year gained. All studies included survival gains in their analyses.

Eight studies described the budget impact of an orphan drug. Most studies (n = 6) only described the costs of treatment itself, but the budget impact study on agalsidase α and agalsidase β as published in the same paper examined the impact of the interventions on the health care budget as a whole, including (substitution of) costs of alternative treatments and other health care usage.

## Discussion

This study reviewed the available evidence for 11 orphan drugs listed on the Dutch policy rule on orphan drugs. In total, over 3,612 studies on these orphan drugs were reviewed, of which 3,274 (91%) were excluded; 338 studies remained. The vast majority of these studies focused on drug effectiveness. These results resemble the results of a recent study in oncologic orphan drugs, which also showed the remarkable absence of pharmaco-economic evidence in these drugs [7].

A notable finding in this review was that interventional studies were available for all of the 11 orphan treatments investigated. In fact, a placebo-controlled double blind randomized trial was available for seven of these drugs. These findings correspond with the results of two earlier studies examining EMA drug approval dossiers, which found that about 60% of orphan drugs were assessed using an RCT [12,13]. While this seems to contrast with the claim that randomized controlled trials are not feasible for orphan drugs [4,5], the current review also showed that the design of these trials differed compared to ordinary RCTs, with a particular focus on multi-center, international studies. Earlier studies comparing orphan and non-orphan cancer and neurological drugs also showed adaptations in study design in orphan drug studies [14,15]. Studies in various therapeutic areas have shown that the size of study population seen in orphan drug RCTs is also significantly smaller than those for non-orphan drugs [14,16].

An adequate study design in studies assessing orphan drugs is not only important because enlisting patients in badly designed studies is unethical [17], but also, due to the small number of patients, there is generally only ‘one shot to do it right’; no treatment-naïve patients might be found after an initial study has been performed. Evaluation of the included studies on effectiveness showed that adaptations to study design were not simply made because of the small number of patients, but also because of other aspects. For instance, in the absence of an alternative treatment it can be considered unethical to withhold treatment from patients [18]. This was especially seen as a hurdle against setting up a placebo-controlled trial for children and infantile patients with high mortality risks (for instance [19]). In addition, when the drug was already commercially available it proved to be difficult to initiate a placebo-controlled trial.

A large part of the studies that were assessed were observational studies. In reading this review, it must be considered that observational studies, when adequately set up and performed, need not always be inferior to randomized clinical trials or systematically overestimate treatment effects [11,20]. Although an RCT is the most appropriate study design to prevent the treatment effect from being biased, in some cases, RCTs might not be a feasible method to study effectiveness of therapy [21]. In this sense, for example, problems relating to the feasibility of blinding or randomization, and ethical considerations also determine whether an RCT is the most suitable study design.

A large share (38%) of full-text publications were excluded because they only provided a summary of available evidence and did not contribute any new knowledge on the particular orphan drug. This high frequency of summaries might be explained by the orphan status of the diseases. Due to the rarity of the diseases, most clinicians might encounter only one or two patients with a particular orphan disease during their careers [22]. This can lead to delays in diagnoses and even misdiagnosis [23,24]. To educate other clinicians about the disease, experts on particular orphan diseases might frequently summarize the current state of knowledge in summary articles. Any delay in making the right diagnosis is unwanted, especially because for some orphan diseases effectiveness of treatment is dependent on timely initiation [25-28]. In addition, a substantial proportion of studies assessed (39%), was excluded because these were not classified as research articles, but had another format (predominantly conference abstracts). In this sense, conference presentations are also hypothesized to be used to disseminate knowledge to other physicians about new therapies and educate other clinicians about orphan diseases.

### Limitations of the study

As with any systematic review, the existence of a publication bias cannot be ruled out – studies without significant therapeutic effects may not be published. Publication bias might especially be prominent for cost-effectiveness analyses. Because of high costs, orphan drugs are rarely cost-effective under conventional thresholds [29] and this might decrease pharmaceutical companies’ incentives to publish such analyses. Publications describing budget impact might be less prone to publication bias, since budget impact is generally small for an orphan drug due to the small numbers of patients treated. However, for all orphan drugs combined, the budget impact is substantial [30]. A publication bias for budget impact analyses can therefore not be ruled out.

The Dutch policy rule was specifically designed to provide early access to high-priced orphan drugs (with an expected budget impact of at least €600,000) for diseases with high unmet medical need. The drugs listed on the policy rule might not be representative for other orphan drugs, which might limit the generalizability of our results to other orphan drugs. Furthermore, transferability of the findings for these drugs to outpatient drugs needs to be studied in future research. However, this review covered 11 inpatient orphan drugs from a broad spectrum of disease areas. The overall conclusions could therefore be considered to represent the situation with orphan drugs in general, and not simply the situation with a specific disease.

### Implications

In assessing the effectiveness of orphan drugs, policy makers can expect an international interventional study to be available. However, policy makers should not expect that country-specific RCTs have been carried out. In assessing the cost-effectiveness and budget impact of treatments, policy makers should not expect to find large body of evidence in the literature. Instead, they are restricted to evidence submitted by pharmaceutical companies or to coverage-with-evidence-development schemes.

Whether the available evidence is considered to be sufficient fully depends on the role of evidence based medicine in reimbursement decisions. Further research is needed to examine the relation between available evidence and positive reimbursement decisions.

## Conclusions

The results of this review showed that at least one interventional study was conducted for all orphan drugs and that at least one randomized placebo-controlled, double-blind, clinical trial was available for over 60% of orphan drugs. The claim that RCTs are not feasible in orphan diseases therefore does not seem to hold for all orphan diseases. Cost-effectiveness and budget impact analyses for orphan drugs are seldom published.

## Competing interests

The authors declare that they have no competing interests.

## Authors’ contributions

TK performed the literature searches. TK and CdS performed the inclusion and exclusion of studies. All authors were involved in the design of the study and have been involved in writing and revising the manuscript. All authors have given final approval of the final version of the manuscript.
